# Sublingual administration of atropine eyedrops in children with excessive drooling – a pilot study

**DOI:** 10.1111/ipd.12219

**Published:** 2015-12-27

**Authors:** Johanna Norderyd, Jonas Graf, Agneta Marcusson, Karolina Nilsson, Eva Sjöstrand, Gunilla Steinwall, Elinor Ärleskog, Mats Bågesund

**Affiliations:** ^1^ National Oral Disability Centre for Rare DisordersThe Institute for Postgraduate Dental EducationJönköpingSweden; ^2^ CHILD, Swedish Institute for Disability ResearchJönköping UniversityJönköpingSweden; ^3^ Department of Otorhinolaryngology and Department of Clinical and Experimental MedicineLinköping UniversityLinköpingSweden; ^4^ Department of Dentofacial OrthopedicsMaxillofacial UnitLinköping UniversityLinköpingSweden; ^5^ Habilitation CentreRyhov County HospitalJönköpingSweden; ^6^ Department for Child and Youth HabilitationCounty Council of ÖstergötlandLinköpingSweden; ^7^ Department of Oral & Maxillofacial SurgeryLinköping University HospitalLinköpingSweden; ^8^ Centre for Orthodontics and Paediatric Dentistry and Department of Medical and Health SciencesLinköping UniversityLinköpingSweden

## Abstract

**Background:**

Drooling can be a severe disability and have high impact on daily life. Reversible treatment is preferable.

**Aim:**

To analyse whether sublingual administration of atropine eyedrops is a useful reversible treatment option for severe drooling in children with disabilities.

**Design:**

The study had a prospective, single‐system research design. The participants served as their own controls. The study period was 3 weeks without treatment, 4 weeks with atropine eyedrop solution 10 mg/mL one drop a day followed by 4 weeks of one drop twice a day. Parents’ rating of their child's drooling was assessed on a 100‐mm VAS, and unstimulated salivary secretion rate measurement was performed together with notations about side effects and practicality.

**Results:**

Parents’ VAS assessment of drooling decreased from a median (range) of 74 (40–98) at baseline to 48 (18–88) (*P* = 0.05) and 32 (12–85) (*P* = 0.004) after 4 weeks of atropine once a day and another 4 weeks of atropine twice a day, respectively (*n* = 11). Unstimulated salivary secretion rates decreased from baseline to end of study (*P* = 0.032). Several parents complained about difficult administration. No irreversible side effects were noted.

**Conclusions:**

Sublingual atropine eyedrops may be an alternative for treatment of severe drooling in children with disabilities.

## Introduction

### Drooling

Drooling is considered to be a normal condition until the age of 3 years, commonly associated to the eruption of teeth^1^. After this age, drooling should cease in children with typical development. Drooling is common in both children and adults with disabilities such as cerebral palsy and other neurological conditions with a prevalence of sometimes up to 30–40% depending on disorder^2^, ^3^, ^4^, ^5^. The aetiology of drooling is mainly associated with oral‐motor dysfunction, such as swallowing incapacity and sensory dysfunction combined with unfavourable body posture, and rarely with hypersalivation^6^, ^7^, ^8^, ^9^.

### Consequences of drooling

Drooling may lead to psycho‐social and physical consequences and can be considered a severe disability in itself with high impact on daily life for the child and family^7^. Facial chapping and skin infections as well as dehydration are some of the physical effects seen in children with severe drooling^10^, ^11^. Frequent changes of scarves, bibs and clothes dampened by saliva are a burden for the household, and the drooling can also damage surrounding objects, such as furniture and computers^6^, ^9^. Psycho‐socially, drooling has been found to have negative influence on social interaction and self‐esteem in children with cerebral palsy. After treatment leading to less drooling, social contacts with peers have been found to increase^12^.

### Treatment

The most common drooling treatment modalities are orofacial regulation therapy^13^, ^14^, drug therapy^15^ and surgery^16^. Other less used interventions may be different modes of bio‐feedback and acupuncture^7^, ^17^. According to a Cochrane review from 2012, there is not sufficient evidence for the effectiveness and safety of any interventions to diminish drooling in children with cerebral palsy^18^.

Surgical approaches when treating drooling differ, but they are all irreversible and come with a variety of risks, such as aspiration and dental caries^19^, ^20^. Medical management aiming at reducing salivary secretion also comes with side effects, but these side effects are reversible. Several studies have presented good outcomes from injections of botulinum toxin A into salivary glands^15^, ^21^. In children, this procedure, however, would most often demand general anaesthesia and some severe side effects have been reported^22^, ^23^. Hyoscine patches and pills have been used, but the efficacy has varied and unwanted side effects are frequent^24^, ^25^. Some promising results from interventions with other anticholinergic drugs have been presented^26^, but there is a need to further explore reversible alternatives which are simple to use in the management of drooling. According to published case reports about adults, sublingual atropine drops have mainly been used for the treatment of clozapine‐induced sialorrhea^27^, ^28^. In a randomized controlled study in adults with cancer receiving palliative care, no difference between atropine and placebo was observed^29^. Atropine is well‐known for dilating the pupils, increasing heart rate, and reducing salivation and other bodily secretions. With the exception of a case report^30^, to our knowledge the use of sublingual atropine eyedrops against non‐medically induced drooling has not been evaluated in children with disabilities.

### Aim

The aim of this study was to analyse whether the use of sublingually administered atropine eyedrops is a useful treatment option for the control of drooling in children with disabilities.

The hypotheses were that sublingual administration of atropine eyedrops is as follows:

1. effective against drooling as evaluated by parents using a 100‐mm visual analogue scale (VAS).
2. reducing salivary secretion as evaluated by salivary secretion measurement.
3. not connected with severe undesired side effects.
4. practical to use as evaluated by the parents.

## Material and methods

Children and adolescents with disabilities 5–18 years of age with a history of excessive drooling were invited to participate in the study after written informed consent from parents and when possible also from the child. Inclusion criteria were children belonging to group 1–2 (healthy or minor disease) according to the ASA (American Society of Anaesthesiologists) Physical Status Classification System^31^ with no allergy to atropine eyedrops or other medical conditions contraindicating the use of atropine. The invitation to participate in the study was made by medical doctors meeting children from outpatient settings for other reasons than drooling. We have therefore no information about how many invited children that primarily declined to participate. Children with untreated dentine caries and insufficient oral hygiene were excluded from the study as they were considered to have an increased caries risk if their salivary secretion would decrease. The study was originally planned as a pilot to a larger study regarding atropine and botulinum toxin type A against drooling in both children and adults, with 10 adults and 10 children as study subjects. After reports of unwanted serious side effects to botulinum toxin^22^, it was decided to solely go forward with the present clinical atropine study including children. There were no available data as base for a power estimation.

**Figure 1:**
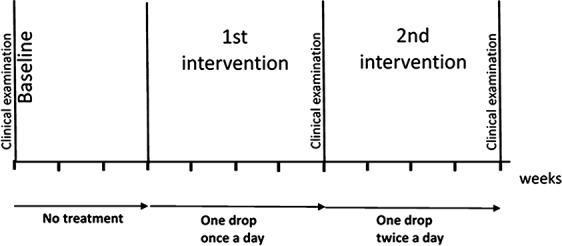
Time schedule of the study. Sublingual administration of atropine eyedrop solution 10 mg/mL during the first‐ and second‐intervention periods. Salivary secretion measurements and parents’ rating of drooling were performed at baseline, after first intervention, and after second intervention.

The study was set up as a prospective, single‐system research design. The participating children served as their own controls. The study design is presented in Fig. 1. The first visit comprised a dental examination and thorough information about the study procedure. Oral‐motor function was assessed using the Nordic Orofacial Test–Screening (NOT‐S)^32^. The study started with 3 weeks without treatment followed by 4 weeks of treatment with sublingual administration of one atropine eyedrop 10 mg/mL in the morning and finally 4 weeks of treatment with one atropine eyedrop twice a day (morning and midday). The total study period for each child was eleven weeks. Visits to the dental clinic were scheduled at baseline, after 7 weeks, and after 11 weeks. During the visits at the dental clinic, unstimulated whole saliva was collected with cotton rolls, which were weighed before and after the saliva collection. The procedure was performed twice at each of the three appointments at the dental clinic. The mean value of the two measurements of saliva from each separate appointment was used for further analysis. The parents also rated their child's drooling on a 100‐mm VAS at each of the three appointments.

Notations were made at home by the parents about any side effects thought to be caused by the atropine eyedrops and continuous free comments regarding practical issues of the treatment.

### Data analyses

Data analysis was undertaken using IBM© spss© Statistics version 21 (SPSS Inc., Chicago, IL, USA) and Stata/MP (version 12.1, StataCorp LP, College Station, TX, USA). Descriptive statistics were used to describe the study population. Wilcoxon signed‐rank test and paired *t*‐test were used for statistical analyses of VAS and salivary secretion rates, respectively.

The study was approved by the Swedish Medical Products Agency (EudraCT No 2007‐003017‐14) and the Regional Ethical Board, Linköping University, Linköping, Sweden (Dnr M89‐07).

## Results

Twenty‐six children (17 boys, 9 girls) agreed to participate in the study. Three (two boys, one girl) left the study directly after the first visit and were considered dropouts together with another four (three boys, one girl) who terminated their participation after finishing the period of one drop a day. Nineteen children (12 boys, 7 girls) completed the study. The mean ± SD age of the 23 participants entering the study was 11.6 ± 4.7 years (median 13, range 5–18). All participants had disabilities with a wide spread of diagnoses as presented in Table 1.

**Table 1:** Main diagnoses of the participants and their oral‐motor function according to the results from the Nordic Orofacial Test–Screening (NOT‐S) assessment

	Children entering the study (*n* = 23)	Excluded children (dropout or incomplete registration (*n* = 12)	Final study group (*n* = 11)
Cerebral palsy	11	5	6
Rare syndromes (e.g., Angelman syndrome)	5	2	3
Other conditions (e.g., Down syndrome, autism spectrum disorders)	7	5	2
NOT‐S mean ± SD (range)	8.1 ± 1.9 (3–11)	8.1 ± 1.4 (6–10)	8.2 ± 2.4 (3–11)
Multiple disabilities	19	10	9
Epilepsy	17	9	8
Intellectual disability	16	7	9

Mean ± SD score (range) for the children with cerebral palsy was 8.0 ± 2.3 (3–11), for the children with a rare syndrome 7.3 ± 1.7 (5–9), and for the children with other conditions 8.9 ± 1.1 (7–10). NOT‐S data were missing from one child with a rare syndrome in the final study group.

The NOT‐S scores are listed in Table 1. All children had tried at least one other alternative to manage drooling before entering the study.

**Figure 2:**
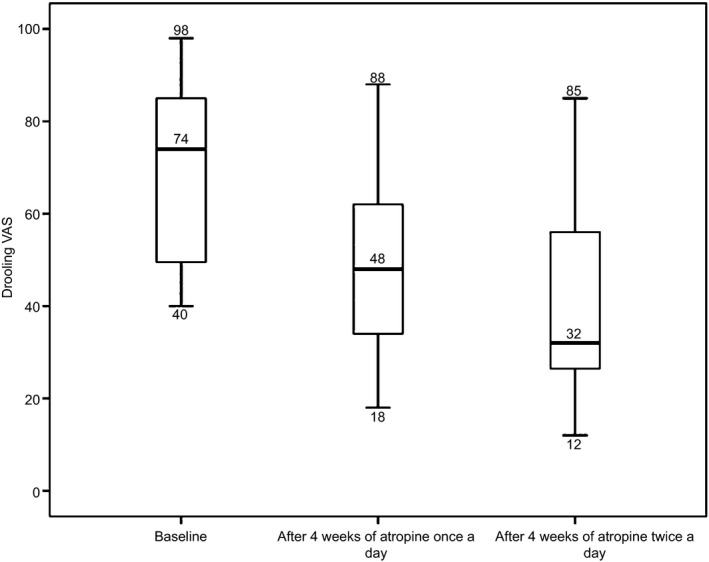
Parents’ subjective assessment according to a 100‐mm VAS regarding their child's drooling at baseline (mean 67.8 ± 21.1) and following 4 weeks (1st intervention) intraoral sublingual administration of atropine (10 mg/mL) with one drop in the morning (mean 50.9 ± 22.4) and after another 4 weeks with one drop in the morning and one in midday (2nd intervention) at the end of the study (mean 41.1 ± 21.8). The median subjective assessment of drooling differed significantly from baseline to after 1st intervention (*P* = 0.05), from after 1st intervention to after 2nd intervention (*P* = 0.026), and from baseline to after 2nd intervention (*P* = 0.004) (*n* = 11).

**Figure 3:**
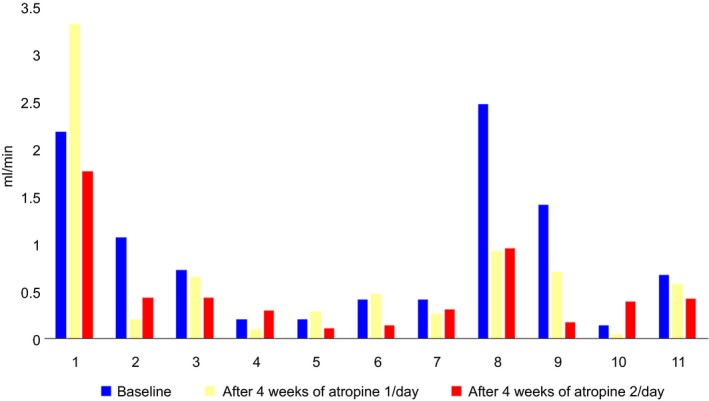
Unstimulated salivary secretion rate (USSR) measured in mL/min at baseline, after 4 weeks with the use of one drop daily, and after another 4 weeks with two drops daily at the end of the study for the 11 children in the final study group. The USSR decreased significantly from baseline to the end of the study (*P* = 0.032).

Three children could not cooperate to the salivary measurement procedures, three children had not complete VAS registrations, and two children had incomplete registrations regarding both saliva secretion rates and VAS. Thus, complete evaluations were only registered in eleven children (seven boys, four girls) with a mean age of 11.8 ± 4.4 years. The parents’ VAS assessment of drooling (Fig. 2) and the unstimulated salivary secretion rates (Fig. 3) are presented for these 11 children.

The self‐reported side effects are presented in Table 2. No irreversible side effects were noted. Three children showed changes in their behaviour: one 13‐year‐old girl showed more anger, one 8‐year‐old boy stopped chewing on his clothes and ceased putting his hands in his mouth, and one 8‐year‐old boy became quiet and withdrawn. These changes disappeared when they stopped using the atropine drops.

**Table 2:** Parents’ reported adverse reactions during their child's sublingual use of atropine eyedrops in the final study group, the children who fulfilled the study with incomplete data registrations, and those who dropped out of the study after 4 weeks of atropine once a day

Adverse reactions	Final study group (*n* = 11)	Participants with incomplete data registrations (*n* = 8)	Participants who dropped out after 4‐week intervention (*n* = 4)
Extensive dry mouth	3	2	2
Miction problems		2	1
Obstipation		2	1
Changed behaviour	1	1	1
Swallowing difficulties			
Tiredness			1
Chapped lips			1
Rosy cheeks			1
Eats less		1	
Increased drooling at end of study		2	
Swollen fingers		1	
Thirst	1		

Parental comments are presented in Table 3. While most families expressed a wish to continue giving their child sublingual atropine on a daily basis or restricted to special occasions, others did not find it more useful than any other treatment options.

**Table 3:** Free comments from the parents of the 23 children entering the study

Positive	Negative
One drop in the morning works well until lunchtime	Difficult with drop size
Two to three times less change of wet bibs and clothes	Dosage of one drop difficult
For the first time we put the clothes in the washing machine because they are dirty, not only wet	Bitter taste not appreciated by the child
School personnel wishes to continue with the drops	Difficult with administration
Works better in the morning than in the afternoon	Becomes too dry when administered twice a day
Perfect to use in social activities	Effect was better in the beginning of study
Better effect when atropine is given before getting out of bed in the morning	Worse drooling after approx. 6 h
	Reduced effect if atropine is administered after getting out of bed in the morning

## Discussion

Sublingual administration of atropine eyedrops to cease drooling in children with disabilities showed promising results.

Families with children with disabilities are often pressed for time^33^ which may have contributed to some dropouts during the study.

As all participating children had been drooling for a long time before entering the study, it was not considered necessary to have a longer baseline period than 3 weeks.

Previously published studies of children with different diagnoses have used the NOT‐S^5^. The NOT‐S data in our study were used to describe the severity of the oral‐motor dysfunction in the studied group of children with disabilities. The very high NOT‐S scores found in our study show that most children had severe oral‐motor impairments in all NOT‐S functional domains as compared to published population‐based data of other children^5^.

Respecting the severity of oral‐motor dysfunction in the study group, it cannot be expected that one single mode of treatment is sufficient and, as has been pointed out by other authors, managing drooling in children with disabilities most likely calls for multidisciplinary measures^11^, where intraoral atropine may be one.

Regardless of whether atropine was administered one or two times per day, a positive effect was reflected in the parents’ assessment of their child's drooling on the VAS. This confirmed our first hypothesis that sublingual administration of atropine eyedrops is effective against drooling.

From the free comments, however, some parents stated that after several weeks of atropine use and less drooling, the drooling increased again towards the end of the study. This should be considered before long‐term treatment is recommended. The parental comments (Table 3) indicate the presence of individual variation in the response to medication with intraoral atropine drops against drooling. We could not find any similar studies of non‐medically induced drooling in children with disabilities published to compare with our results. Rapoport^30^, however, reports a case of a 14‐year‐old boy receiving palliative care who was given sublingual atropine drops every 6 h for treatment of severe drooling. The boy's family and healthcare givers thought the atropine treatment to have a fast onset, to be well tolerated, and that it decreased the boy's sufferings.

As expected, atropine had a reducing effect on the salivary secretion rate. The second hypothesis that sublingual administration of atropine eyedrops is reducing salivary secretion was confirmed.

The measurements carried out in this study were only partly standardized in the way saliva was collected and measured. Time of day, time after atropine administration, and time after food intake differed greatly between study subjects. Such factors are known to influence the salivary secretion rate^34^. There is also missing data from the children that were not able to cooperate to the measuring procedures and therefore were excluded from the study. The results regarding salivary secretion rate should therefore be considered with caution.

Reduced salivary secretion rate in itself is from a dental view undesired as decreased salivary secretion rate increases the risk for dental caries and other unfavourable oral conditions^19^, ^20^, ^34^. It is therefore important that anyone who is planned for a treatment causing dry mouth should be examined by a dentist before the treatment is administered to assess the oral health and provide necessary measures to avoid oral complications. In addition, children with severe disabilities most often have several assisting persons (e.g., parents and personal assistants) taking care of their oral health. It is therefore important to inform and instruct these assisting persons about the particular needs related to the oral health of the individual patient.

All participants received the same dosage of atropine regardless of age and body weight. This can explain the cases of extreme dryness of mouth, which was the most common undesired side effect reported. The advantages with atropine drops as compared to some other medications for drooling are that the method is cheap, accessible and has a fast onset. It was also found to have a comparably short effect. That the effect of dry mouth is short can be seen both as negative and positive. In most cases, the short duration should be positive for the patient, as it gives possibilities for modification of the dose.

The self‐reported changes of behaviour were the most severe side effects, but we find it difficult to assess whether the changed behaviour in individual cases actually was caused by the atropine or could be explained by other external or internal factors. We therefore accepted the third hypothesis that sublingual administration of atropine eyedrops is not connected with severe undesired side effects. It should be noted, however, that vital signs such as heart rate, blood pressure, and oxygen saturation was not registered in this study. In the report by Rapoport^30^, no obvious adverse reactions were observed.

The atropine eyedrops are not produced for intraoral use, and this can probably explain the reason behind some free comments from parents about difficulties associated with dosage and administration. A ready‐made oral solution had been desirable, both for practical and prescription purposes. The fourth hypothesis that sublingual administration of atropine eyedrops is practical to use was rejected.

The information that some parents had assessed a decreased effect on drooling towards the end of the study is interesting. Most likely, the submandibular glands were the ones most affected by the atropine and it cannot be ruled out that compensatory saliva production started from the parotid glands. Such compensatory salivary production has been found in animal studies^35^.

Some parents stated that they were not as much bothered by their child's drooling as school personnel and assistants were. This made atropine treatment in the morning more important than treatment in the afternoon and may be the reason why some of the study participants left the study before the final evaluation after 11 weeks.

The comment that atropine had better effect if administered before the child got out of bed in the morning is probably related to the lower salivary secretion rate during the night. When salivary secretion increases during the morning hours, a larger amount of the atropine is expected to be washed out of the oral cavity by drooling.

Single‐system research design is useful in development of new intervention models, but there are obvious limitations in the design of this study, threatening its validity. There were no placebo controls or blindness procedure, the study time was short, and the number of subjects were limited. This, together with heterogeneous diagnoses of the participants, makes further studies needed to evaluate the effects of long‐term use of intraoral sublingual atropine drops for treatment of excessive drooling. There may also be an interest for other areas of dentistry to study short‐term use of intraoral atropine drops to facilitate procedures in patients with high salivary flow.

In conclusion, intraoral sublingual administration of atropine eyedrops decreased drooling and reduced salivary secretion rate in children with disabilities. A majority of the parents considered the method to be efficient but sometimes not practical. No irreversible side effects were noted.

**Why this paper is important to paediatric dentists**

- Sublingual administration of atropine eyedrops could be considered as an option for treatment of excessive drooling in children with disabilities.

## Conflict of interest

The authors declare no conflict of interest.
